# The Ethical Foundations of Accompanying Patients: The Need for Institutionalization

**DOI:** 10.1007/s11673-025-10482-z

**Published:** 2025-12-01

**Authors:** M. Shankland, A. Ferrand, I. Ganache, M. -A. Côté, M. -P. Pomey

**Affiliations:** 1 Bioethics Program, Department of Social and Preventive Medicine, School of Public Health, Université de Montréal, 7101 Av. du Parc, Montréal, QC H3N 1X9 Canada; 2 Pragmatic Health Ethics Research Unit, Montreal Clinical Research Institute (IRCM), Université de Montréal, 110 Av des Pins O, Montréal, QC H2W 1R7 Canada; 3 Accompanying Patient, Independent Researcher, 900 R. Saint-Denis, Montréal, QC H2X 0A9 Canada; 4 CHUM Research Centre, Health Innovation and Evaluation Hub and Centre d’excellence sur le partenariat avec les patients et le public, 900 R. Saint-Denis, Montréal, QC H2X 0A9 Canada; 5 Department of Health Management, Evaluation and Policy, School of Public Health, Université de Montréal, 7101 Av. du Parc, Montréal, QC H3N 1X9 Canada

**Keywords:** Accompanying patient, Patient advisor, Patient partner, Patient expert, Patient navigator, Patient partnership, Public health, Public health ethics, Ethics, Ethical foundations

## Abstract

The partnership approach is part of a broader shift in healthcare that seeks to empower patients in their own care. Accompanying patients (APs) extend the concept of peer support. They are experienced patients who engage directly and individually with new patients, enhancing the partnership model. However, the ethical foundations of this role and the ethical tensions arising from its integration are underexplored. To identify the key skills and values required by APs and the ethical tensions they experience, we conducted a qualitative study based on in-depth interviews with nineteen APs between January and May 2021 in Québec, Canada. Interviews explored motivations, skills, values, and experiences in healthcare institutions. Thematic analysis was complemented by Tannahill’s public health ethics framework to interpret the ethical tensions identified. The core values identified for this role were empathy, respect, and altruism. All participants agreed that experiential knowledge was paramount. APs identified independence from healthcare institutions as the key to connecting and developing a trusting relationship with patients. However, they also expressed a need to be part of the healthcare system to ensure the sustainability of their role. Our results show an ethical tension between the philosophical core of APs and their needs to pursue their functions. We conclude that independence and authenticity as an AP is not necessarily incompatible with institutionalization, professionalization, and remuneration. These can be achieved if AP programmes are aligned with the philosophy of patient partnership, structured with flexible criteria, and remain self-regulated.

**Supplementary Information:**

The online version contains supplementary material available at 10.1007/s11673-025-10482-z.

## Introduction

Over the last two decades, the relationship between healthcare providers and their patients has shifted from a more paternalistic model to a patient-centred model and, more recently, to a partnership model (Dumez and Pomey 2019). This evolution reflects growing recognition of the experiential expertise of those receiving care (Bouabida et al. 2021; M.-P. Pomey et al. 2016; Richards et al. 2023). Patients navigating the healthcare system acquire expertise, interpersonal qualities, and know-how that complement healthcare providers’ scientific and professional knowledge. In this position, they can become fully integrated healthcare team members, making decisions about their own health, medical, and social conditions with the help of healthcare providers who provide information and guidance (Krist et al. 2017; Vigneault et al. 2015; Vahdat et al. 2014). This partnership approach empowers individuals to act as collaborators in their healthcare. Some individuals may extend this by training healthcare providers, conducting research, or participating in healthcare governance (Pomey et al. 2016; Martin and Finn 2011; Liang et al. 2018).

Internationally, peer support programmes have been established in the fields of oncology (Gabitova and Burke 2014), cardiology (Winder et al. 2004; Colella and King 2004), neurosurgery (McPeake and Quasim 2016), transplantation (Guha et al. 2020; Taha et al. 2022), amputation (Reichmann and Bartman 2018), and burns in intensive care (Won et al. 2021; Badger, Acton, and Peterson 2017). In Québec, Canada, the introduction of peer support workers has primarily focused on mental health (Walde et al. 2023; Gates and Akabas 2007) but the initiative has been extended to other healthcare sectors (Won et al. 2021; Badger, Acton, and Peterson 2017; Pomey, Paquette, et al. 2023; Gabitova and Burke 2014; Winder et al. 2004; Colella and King 2004; McPeake and Quasim 2016; Guha et al. 2020; Taha et al. 2022; Reichmann and Bartman 2018). This expansion has led to the PAROLE project (Patients Accompagnateurs une Ressource Organisationnelle comme Levier pour améliorer l’Expérience des patients [The Accompanying Patient, an Organizational Resource as a Lever for an Enhanced Patient Experience]) (Pomey et al. 2021). This programme is an organizational resource designed to enhance the patient experience (Pomey, Nelea, et al. 2023).

Patients involved in the healthcare system in this way are called accompanying patients (APs). APs are people who have lived through an episode of care and now share their experience with others who are beginning similar trajectories, providing informational, educational, emotional, and navigational support (Pomey, Paquette, et al. 2023; Shankland et al. 2022; Vigneault et al. 2015; Walde et al. 2023). Their activities take place through in-person meetings, phone calls, or video conferences. In Québec, all APs are volunteers. They cannot receive remuneration but can be reimbursed for travel and living expenses.

There is currently no regulatory framework governing AP involvement in the Québec healthcare system, although collaboration with legal scholars has made it possible to propose guidelines (Boutrouille et al. 2021). A scoping review has identified that ethical principles relevant to APs have yet to be addressed (Bouabida et al. 2021). The literature on patient partnerships has addressed ethical concerns when patients participate in teaching, research, or governance (Bouabida et al. 2021). APs, however, are distinct in their direct involvement with patients receiving care. As interprofessional and interpersonal relationships are becoming more complex, examining the potential ethical issues that APs may encounter is imperative.

This paper has two objectives. First, it seeks to clarify the ethical foundations guiding APs in Québec and how they perceive their roles and responsibilities. Second, it investigates ethical tensions that may arise among stakeholders in organizational settings and explores strategies to resolve them.

## Methods

A qualitative descriptive study was carried out following Thorne’s approach (Thorne 2016) to answering qualitative research questions in practical public health. This approach allows flexibility in working with data derived from experiential and subjective knowledge.

### Participants

All interviewees were enrolled in an already established AP programme implemented in the clinical fields of oncology and chronic conditions, including cardiac conditions, reimplantation, amputation, burn survivors, transplantation, and neurosurgery.

### Data Collection

Convenience sampling was used. The principal investigator (MPP) provided an initial list of potential participants in the PAROLE project and other AP programmes. Two researchers (MS and AF) contacted APs by email to invite participants. Informed consent was obtained from all participants prior to interviews, which took place between January and May 2021.

### Settings

Data was gathered through in-depth interviews lasting between forty-five and seventy minutes. Topics included personal values, AP status, their role within the care team, AP activities, and the organization of meetings (see Additional file 1).

Interviews were conducted by two researchers (MS and AF), either online (using Zoom or Microsoft Teams) or by telephone, according to participant and researcher’s preference. All interviews were audio-recorded with prior consent. The research was conducted in Québec, Canada and received ethical approval from the Research Ethics Committee (17.260) of the Centre Hospitalier de l’Université de Montréal (CHUM).

### Data Analysis

A qualitative thematic analysis was undertaken following the framework method developed by Ritchie et al. (2014) and adapted for health research with a multidisciplinary team by Gale et al. (2013). First, interviews were transcribed and anonymized. Second, a researcher (MS) condensed the descriptive data to gain familiarity with the material (Gaudet and Robert 2018), allowing the synthesis of participant experiences without interpretation.

Following an iterative approach, the research team met to agree on primary codes, drawing on a scoping review of the ethical foundations of APs (Shankland et al. 2022). One researcher (MS) conducted open coding using the predefined codes, then re-coded all transcripts using emerging codes. Given the complexity of identifying values, we used a latent approach, giving us the necessary space to understand the meaning of a statement instead of sticking to words chosen by participants to express their skills and values.

The research team developed a working analytical framework and agreed on four main themes: (i) becoming an AP, (ii) being an AP, (iii) APs in action, and (iv) healthcare system responsibilities. Analysis was further refined by examining gender, age, and type of condition. Gender was recorded according to the participants’ gender, who all fall within the binary spectrum, and age was categorized in ten-year intervals from twenty-five to seventy-four years.

The second stage of the analysis applied the analytical framework to all transcripts. Data were summarized in a spreadsheet that included codes, classifications, and illustrative quotations. Tables were created to assess differences by gender, age, and condition.

For the final stage, Tannahill’s framework (2008) for health promotion, public health, and health improvement informed data interpretation guided the discussion of the ethical tensions identified. This framework incorporates ten values—do good, do not harm, equity, respect, empowerment, sustainability, social responsibility, participation, openness, and accountability—and places significant emphasis on scientific evidence. These are principles that are accepted and well-known in public health ethics studies. Using this framework, ethical tensions were assessed and categorized by stakeholder group (patients, APs, clinical teams, healthcare system, and society) in relation to each value. The analysis was guided by the philosophical core of patient partnership, as illustrated in our results.

## Results

### Description of the Population

Of the twenty-three potential participants contacted, twenty-one agreed to participate, and nineteen completed the interviews. One potential participant died before taking part, yielding a response rate of 82.6 per cent. Table 1 shows the profiles of the participants.

**Table 1:** Participants’ profiles

***Categories***	***Characteristics***	***Number***
Gender	Women	13
	Men	6
Age	25–34 years old	2
	35–44 years old	2
	45–54 years old	3
	55–64 years old	8
	65–74 years old	4
Type of disease or condition … … Cancer	Breast cancer	5
	Gyneacological cancer	1
	Hodgkin lymphoma	1
	Metastatic cancer	1
	Oncogenetic trajectory	1
… Chronic	Cardiac condition	1
	Reimplantation	2
	Amputation	1
	Burn Survivor	3
	Transplant	2
	Neurosurgery	1
Years as an accompanying patient	< 1 year	4
	1 to 3 years	4
	3 to 5 years	3
	> 5 years	8

### Becoming an AP

Of the nineteen participants, ten were identified by healthcare providers (physicians, nurses, health coordinators, or clinical administrative assistants) as having the qualities and skills sought in APs and were subsequently recruited. Five participants volunteered directly with their healthcare provider. The Mutual Aid Patient Association identified two volunteers who demonstrated the attributes needed to accompany inpatients undergoing similar experiences. Two additional participants became involved through the recommendation of a family member or friend (see Table 2).

**Table 2:** Becoming an AP

**Selection**	**Definitions**	**Number of participants**	**Quotes**
Professional or hospital employee	Respondents were recruited by their healthcare provider or a patient partnership coordinator.	10	“I met [the researcher] at a conference, and I was talking about the need to do a kind of 101 course for patients who do not know how to navigate the system when they have never been ill, and she came to see me to present the project. I joined the project.”
Volunteer	Respondents volunteered to their healthcare provider or a patient partnership coordinator.	5	“From the very first meeting, when [my doctor] suggested me [AP's service], I told him straight away: ‘I want to do that, I want to do that.’ He calmed me down: ‘We will start by treating you here... (laughs) and we will see about that later, we will see about that later...’”
External organization	Respondents were recruited by or involved in an external patient association to become AP	2	“I was involved with a Mutual Aid Patient Association, and then this was suggested to me, and I found it very interesting to get on board.”
Friends or family	Respondents were recruited by a friend or family member.	2	“My sister wanted to raise money, she wanted to volunteer, and I wanted to get better, to begin with. However, I think it was really my sister who pushed me to get involved.”

In all cases, recruiters conducted an initial screening to assess personality fit for a supportive role and then determined whether the individual would be selected.

Participants described various motivations for becoming involved in the healthcare system. The most common was to give meaning to their own experience of living with a disease or health condition. Thirteen participants reported feeling compelled to act after what they had endured, seeking to transform their health trajectory into a positive experience by becoming an AP. Eight participants wished to give back to the healthcare system and their healthcare providers, while four expressed a specific interest in collaborating with their healthcare team. One participant reflected on the ethical implications of being recruited directly by their doctor: “I cannot really say no to that! Say, no, I could not do that. There is a bit of an ethical matter here. It is like, ‘Well, yes! If I can help science and help people or … there is no problem, obviously.’”

Other motivations included having free time (*n*=3) and wanting to help (*n*=3) or to give hope (*n*=1) (see Table 3).

**Table 3:** Motivations for becoming an AP

**Motivations**	**Definitions**	**Number of participants**	**Quotes**
Personal	Respondents mentioned personal interests in being an AP.	13	“I needed meaning in my life. Disease had changed the course of my life a lot (laughs). Many things suddenly made me lose all my bearings, and now it is like, ‘Phew, what am I going to do with this?’ Simply put, I have lost my job due to my three cancers. You know you are a bit of a wreck. You cannot... Okay, yes, you have fought a lot, and now life has given you this. However, what do I do with it? I was looking for much meaning.”
Giving back	Respondents wanted to give back to the system and healthcare providers.	8	“I felt the need to do something, and first of all, I felt very, very indebted to my donor, right? Because it was someone who gave me that liver, so I really felt indebted, firstly to my donor, but also the CHUM.”
Professional	Respondents wanted to get involved because of specific healthcare providers or employees.	4	“[My doctor] is wonderful, and I want to clarify that. He is someone you meet and immediately want to invite home for dinner on Sunday evening with his whole family. He is wonderful, not just as a doctor, but as a human being.”
Free time	Respondents had free time (i.e., retired or unemployed).	3	“I was a year away from retirement. I retired in 2013, I knew I had much time coming, and I wanted to keep busy.”
Helping	Respondents wished to help other patients with a similar disease or health condition.	3	“I said, ‘Look, I do not want lonelier women walking alone. Because I felt pampered, I will give back what I had around me thanks to the people I had.’ It was all about giving to others who had nothing.”
Giving hope	Respondents wished to give hope to other patients with a similar disease or health condition.	1	“It was important to testify that you are alive after cancer, that it is not a death sentence. So, I wanted to be living proof.”

Twelve participants mentioned the need to distance themselves from their own healthcare experiences when becoming an AP. Among them, seven individuals emphasized the importance of allowing time to pass between their episodes of care and assuming the role of AP, while five expressed the contrary view. Despite these differences, all participants agreed on the significance of maintaining emotional detachment when interacting with patients, accepting their health condition, and being able to discuss it openly. We noted that most participants who disagreed with the need for distance were individuals with chronic diseases, who are unlikely to ever be able to achieve it. Conversely, most participants who advocated for distance were individuals who had experienced periodic diseases. No other significant differences were noted when data were analysed by gender or age.

### Being an AP

#### Key Skills

Participants identified a range of skills required to serve effectively as APs. All nineteen participants mentioned experiential knowledge as being paramount to their role, and all but one highlighted active listening. Other frequently cited skills were empowering others (*n*=14), providing support (*n*=13), adaptability (*n*=12), reassurance (*n*=11), and resilience (*n*=10). Also cited were support for health democracy (*n*=7), eloquence (*n*=4), and curiosity (*n*=3). Table 4 provides definitions, sub-competencies within these skills, and illustrative quotes.

**Table 4:** Key Skills

**Skills**	**Definitions**	**Number of participants**	**Sub-skills**	**Number of participants**	**Quotes**
Experiential knowledge	Experiential knowledge is acquired through personal experience with a disease or living with a health condition. This knowledge cannot be learned through discursive reasoning. It enables a unique connection with patients.	19	Experiential knowledge	19	“I can really see that the bond is created, the bond is created when we get to the most difficult period of the care episode, and the patient understands a little bit what we have been through, and then we feel like we are getting closer... I can really feel it in the patients’ reactions. They say to me: ‘Finally, someone can understand me, someone has been through the same thing I have.’”
Active listening	Active listening involves asking patients open-ended questions to understand them and their preferences.	18	Active listening	18	“A good listener, able to ask open-ended questions.”
Empowerment	Empowerment is the ability to instil confidence in patients and motivate them to engage actively in their care. It includes becoming a partner in one’s care, navigating the healthcare system and preparing for appointments, and taking back control of one’s life.	14	Empowerment	14	“I tell patients that if you do not agree with your care plan, you have to say so. If you have diabetes, and we tell you to eat this, and you do not want to eat that, you have to say so.”
Support	Support is a positive attitude that aims to ease the burden of care and reshape the perception of life with the disease or health condition, spreading a sense of happiness and optimism, and showing sensitivity to the feelings of others.	13	Support	12	“To be able to share with them the experiences, emotions, and vulnerability that we experience when we have cancer, so for me, that is the most important thing, to support other women who are going through the same thing.”
			Humour	1	“And then have a little touch of humour, not a big one. Look, we are not clowns here! However, just, you know, sometimes, a touch just to feel that there is a little smile going on at the end of the phonecall or sometimes, a burst of laughter, even, you know?”
			Happiness	2	“To be able to walk around the floors, go and see patients and give them a small amount of happiness, [...] something positive, if you make an effort, well you will get results, but [...] it is not a guarantee, because there are some for whom it is always more difficult, it always depends on what kind of accident you have. However, to give a little positivity.”
Adaptability	Adaptability is the ability to personalize interventions to patients’ individual needs, nuancing perspectives and approaches in light of experiential differences.	12	Personalized	7	“So, I think it is really a matter of adapting to everyone's needs, even if it is the first time you have seen them, you know, you have to get to know the person a little bit. Then, at the same time, there are different personalities. For example, of the people I have met, there are some who live completely in denial and think that in three days, they will be out of the hospital, and others are completely destroyed. It is also about accepting the person where they are in their journey.”
			Neutrality	4	“I have had situations where it was not a good experience with healthcare providers, others in which it was magnificent, others in which it was normal and ordinary, but that does not mean I am going to base my support on negative experiences. So, you have to know how to balance things out, and you have to know how to look at the situation in a neutral way.”
			Communication	6	“Be able to communicate. In the depth of her experience and with nuance, I would say, because my experience is unique and the experience of other women is unique too, you have to be capable of nuance too.”
Reassurance	Reassurance means adopting a positive attitude and mitigating the fears of the patient or, when the patient is unconscious or unable to engage, their family.	11	Reassurance	11	“It is a relationship in which you have someone who has been through something, and the other needs reassurance and guidance.”
Resilience	Resilience is the ability to overcome their own difficult, painful, or traumatic experiences and the associated emotions that can resurface while supporting others.	10	Resilience	9	“It also takes a small amount of strength in terms of mental health. You must be a little bit, I would say, not in a weak state because you will not be able to help other people. So, mentally, I think it is important for the person to be strong enough. Resilience.”
			Calm	2	“You have to be calm, very calm, and, as I said, you always have to accept and understand who you are.”
			Personal balance	2	“I think it takes a good personal balance; you must know yourself.”
			Self-acceptance	2	“Self-acceptance, because it is not always easy to talk about yourself and accepting that you have had cancer and all its consequences.”
Health democracy	Health democracy refers to the motivation and skills needed to counteract the disempowering effects of medical jargon and complex health systems. APs foster health democracy by using patient-appropriate communication and by helping patients understand, navigate, and make informed choices about their care.	7	Awareness	2	“The nurse team is like: ‘Well, this gentleman here wants to smoke cigarettes,’ but, after a reimplant, smoking a cigarette, I mean, take your fingers off right away, you know, if it does not work, we will untie them, and you will go home, but you are not going to smoke a cigarette you know, it is over there [...], but I can get into the room, and I tell him exactly that, well, if you feel like smoking, I mean, your fingers, it is over, I promise you, there is no: ‘ah maybe... ’”
			Communication	4	“We discovered just how much, for example, in physiotherapy, occupational therapy, and so on, I could stimulate and motivate. That even things that were sometimes suggested by team members and refused by patients, all I had to do was get behind them and talk about them in my way.”
			Popularization	1	“By saying: 'Well, you know, that is kind of how it is going to be, you know, we are all a little different, but like, you know, these are the steps, if you have questions, well, this means such and such, the doctor tells you such and such, okay well,' there is much popularization.”
			Modesty	1	“What is needed to become an accompanying patient? I think [modesty] is very important [we are] a very ordinary person.”
Eloquence	Eloquence refers to APs’ ability and ease in expressing themselves and discussing their experiences.	4	Eloquence	4	“Of course, when it comes to the profile of accompanying patients, they must be able to express themselves properly.”
Curiosity	Curiosity is the desire to keep abreast of relevant advances in the medical and institutional world to better support patients.	3	Curiosity	3	“You also must develop your knowledge of the disease and be curious. I am curious by nature, and that has helped me a lot because I ask many questions, whether to my doctors or even to my students. I ask many questions when co-leading the workshops because I want to learn. I want to understand how things work.”

### Key Values

Most participants identified empathy (*n*=11), respect (*n*=11), and altruism (*n*=10) as values needed to fill the role and responsibilities of an AP. Also important were humanity (*n*=8), honesty (*n*=7), benevolence (*n*=6), and compassion (*n*=4). Table 5 elaborates on definitions and frequencies and gives examples.

**Table 5:** Key values

**Values**	**Definitions**	**Number of participants**	**Sub-values**	**Number of participants**	**Quotes**
Empathy	Empathy is the ability to share and understand a patient’s emotions by putting oneself in their position.	11	Empathy	11	“I try to get into their shoes, to be empathetic.”
			Comprehensiveness	2	“I am very jovial, someone who can understand another person easily.”
Respect	Respect means treating others as equals, remaining open to different therapeutic choices and avoiding judgment of decisions made by patients, even when those choices are personally or morally challenging.	11	Respect	7	“You need someone who has great respect for the people you are going to meet, great respect to believe in their abilities and be there to support them.”
			Openness	5	“You know, someone who would say, ‘Women need to have surgery, they need to have their ovaries removed, it has got to go!’ you know? You cannot be categorical; you must be open to some of everything.”
Altruism	Altruism is acting selflessly with a focus on the needs of others. Altruism reflects loyalty, dedication, and a patient-centred approach.	10	Altruism	5	“To be there for others and not for yourself.”
			Devotion	8	“Is not to be there for our own needs, in the sense that... […] it is not for our personal battles.”
			Loyalty	1	“I am there for the patients, first and foremost, so I mean, I can, in fact, completely understand and accept and support the position of the family who finds it unacceptable that the patient should be left to scream for so many hours in a hospital.”
Humanity	Humanity refers to recognising the intrinsic value of every person and approaching interactions with sensitivity, warmth, and joy.	8	Humanity	6	“I felt like I was bringing humanity to the workplace, bringing something human, which was really, really good.”
			Sociability	2	“You must like doing social work there, talking to people.”
Honesty	Honesty is telling the truth, having the ability to be trusted, and building trust by avoiding hidden agendas or personal interests in a patient’s condition.	7	Honesty	5	“To have words of encouragement but to be realistic, to be a person who can tell the reality, the real reality, the pain, how difficult chemotherapy is, to be honest, to be able to be transparent.”
			Authenticity	1	“You must be very authentic and act on your instincts.”
			Liberty	2	“It gives us much freedom regarding what we can talk about.”
Benevolence	Benevolence is the desire to ensure the well-being of a patient in need of support.	6	Benevolence	4	“It is benevolence, isn't it? We are here to offer benevolence. That is our role.”
			Generosity	2	“Of course, you have to want to give something back. Of course, it is a gift, but I think that if you do not have that generosity, that drive, you will never get something back.”
Compassion	Compassion is a deep sense of sympathy for the suffering or bad luck of others and a desire to help them.	4	Compassion	4	“Having compassion for people? You know, knowing and trying to understand the situation a little.”

Five motivations were identified for continuing the role of AP: personal gain (*n*=11), helping other patients (*n*=9), peer support (*n*=8), awareness (*n*=1), and medical privilege (*n*=1) (see Table 6). Personal gain can include emotional benefits, so many AP statements reflect both altruistic and self-motivated reasons, highlighting the interplay between intrinsic and extrinsic drivers of care.

**Table 6:** Motivations for continuing to be an AP

**Motivations**	**Definitions**	**Number of participants**	**Quotes**
Personal gain	Personal gain refers to benefits attained by APs, including emotional gratification.	11	“Because it makes so much sense, […] it is very gratifying for me. I find a lot in it. It gives meaning to my life.”
Helping	Helping mean a desire to provide benefits to others.	9	“Well, when I give hope, it gives me great pleasure; there is a great satisfaction in being able to help people.”
Peer support	Peer support refers to support by AP communities that have been created autonomously or organized by the research group.	8	“We meet every two weeks, have a Zoom with all the other accompanying patients who are really nice, and I really think it is a great group.”
Awareness	By *awareness*, we refer to the desire to promote and spread knowledge of the important value added through the AP role.	1	“Because I feel that the sooner, we make healthcare providers aware of the importance of patient partnership, the better.”
Medical privilege	Medical privilege refers to the AP benefit of increased access to medical and care providers.	1	“There is a side, well, I would not say ‘selfish,’ but it gives me an entry point to talk to my doctor, too. You know, I go through my point of contact who is on the team, and then, well, I can ask him a question, and tomorrow I will have an answer.”

### AP Interactions

#### Interactions with Patients

The importance of being a model of hope for patients was reported by eight participants, who emphasized that APs provide living proof that treatments can work. In contrast, four participants highlighted the importance of conveying positivity, while another four focused on the strength and energy transmitted through their interactions rather than on hope itself. These preferences may reflect participants’ personal health trajectories, as some were living with chronic conditions that could not be cured.

The relational dynamics between APs and patients were described in ambivalent terms. Most participants agree that the relationship is one of trust, though framed by professional distance (n=11). The information shared is often of an intimate or sensitive, leading to friendly interactions that fostered closeness and rapport. However, participants were clear that these relationships remained contained within a professional framework (*n*=11).

Two participants noted that they developed a more overtly friendly relationship with patients. The frequency of encounters and the duration of the relationship seem to be determining factors. For example, one AP in neurosurgery explained that they were sometimes at patients’ bedsides for several months, developing a unique relationship with them. Another AP, who was supporting others with cardiac conditions, described sustaining contact with young patients during their transition from paediatric to adult services, providing encouragement to adhere to treatment and attend appointments: Their support takes the form of short exchanges over a long period. They explained: “I replace the parent or the good angel, if you like, the good conscience, saying ‘Well, yes, you have to take the medicine, it is better to buy it!’ or ‘You have to come to the hospital, you have to make the appointments, even if you feel well.’”

In both scenarios, APs established friendly relationships with the patients they met because their relationships occurred over a more extended period or with greater frequency. Conversely, APs in oncology attend to patients at pivotal times during their healthcare journey, but within a limited time frame, limiting the formation of deeper sustained relationships.

One participant also noted the challenges of blurred boundaries. They accepted friendly requests from a patient but found themselves uncomfortable when the requests became intrusive and did not know how to return to a professional relationship without disengaging the patient in their treatment.

#### Interaction with the Clinical Team

The level of collaboration between APs and clinical teams varied. Most participants (*n*=14) had a positive overall experience with clinical teams, with ten reporting exclusively positive encounters. APs highlighted the reciprocal exchange of information as central to effective collaboration: clinical teams provided updates on patients’ health status, while APs relayed socially significant observations (e.g., patients’ misunderstanding of clinical advice leading to harmful behaviours). Participants also appreciated the possibility of reporting their encounters with patients to healthcare providers by talking to them or sharing notes. These participants expressed a sense of usefulness, belonging to the clinical team, and positively impacting the unit.

In contrast, five participants reported exclusively negative experiences with the clinical team, including a lack of understanding of the AP role and being unknown to or ignored by the team. This lack of understanding led to healthcare providers’ resistance to AP integration into the care process. Participants discussed psychologists’ and social workers’ feelings of replacement and physicians’ suspicions. Two of them specifically mentioned feeling ignored and out of place. The indifference shown by healthcare providers was reported as leading to misunderstandings. For example, one participant shared their frustration at traveling to the hospital only to find out that their services were impossible because of painkillers administered to the patient shortly before their visit. Even though the participant understood the need for painkillers, they said they could have been told to postpone their visit. These two participants belonged to an external organization and were not directly involved in a team. However, it is worth noting that another participant from the same organization as the two above had only positive encounters. This was likely due to this participant, over time, becoming more directly involved with the institution, while a third party organized hospital visits for the other two.

## Healthcare Institutions’ Responsibilities Toward APs

### Need for Support

Participants identified various forms of support that they appreciated or wanted for carrying out their duties. Collaboration between health professionals and APs through a facilitator was seen as critical (*n*=8). Healthcare providers were expected to refer patients to APs’ services and, in inpatient settings, to guide APs toward which patients to visit. Access to institutional psychologists or social workers was valued following particularly challenging encounters (*n*=5). Coordination of AP activities was sometimes experienced as complex (*n*=4). Participants recommended that institutions designate a resource staff member to oversee AP activities, act as a liaison with the clinical team, match APs with patients, and provide feedback on the impact of their involvement. Such a role was described as a driving force for programme sustainability. Integration into the clinical unit was also considered an important support mechanism (*n*=4). Introducing the AP to the unit where they will work is a good starting point for offering their services. Finally, some participants emphasized the value of peer connection, expressing a wish to be in contact with other APs to share experiences (*n*=3).

### Type of Recognition Valued or Wished For

Participants identified various types of recognition valued or wished for. Being included in the healthcare system on an equal footing with healthcare providers and staff was the most common form of recognition (*n*=12). Promoting the role of APs to the public, members of the healthcare community, and patients experiencing a disease or health condition was also a recurring theme (*n*=11). Other types of recognition identified by participants were compensation for expenses incurred (*n*=8), guidance (*n*=6), acknowledgment (*n*=4), and acknowledgement of experiential knowledge as expertise (*n*=4) (see Table 7).

**Table 7:** APs’ Recognition

**Recognition**	**Definitions**	**Number of participants**	**Quotes**
Inclusion	Be invited to employee appreciation events, such as holiday parties. Be included in benefits usually reserved for employees, such as flu shots. Receive relevant communications that impact their services, such as manager changes and unit news, and have an access card allowing them to move freely on the floors where they provide services.	12	“Integrate the patient into the team where he or she will be working so that he or she receives relevant emails about management changes, staffing changes, and new developments in the unit. Give them a card.”
Known	Promote APs to all community stakeholders, even as separate from other volunteers, and introduce them to the clinical team.	11	“I think this is basic: knowing who you are working with. Introducing the patient to the team and the team to the patient.”
Compensation	Receive compensation for travel (e.g., fuel or public transport), parking, and meals when services require at least a half day’s presence.	8	“Of course, if we can come up with some kind of compensation that at least recognizes that you have come all this way, that it takes a little bit of money and a little bit of effort, we will try to do what we can with what we have to offer.”
Guidance	Receive ongoing training, supervision and support.	6	“It is the training that we have to give ourselves, the giving of the real fundamentals, the giving of the real values.”
Acknowledgment	To be greeted and thanked.	4	“I get comments like: ‘Ah, thank you!’ The people around me, […] I get a pat on the back from them, that is enough for me.”
Expertise	Recognize experiential knowledge by soliciting their opinions and inviting them to speak publicly on an issue related to their role.	4	“I have done several presentations, and I am always invited along with the team when there are presentations at any level.”

### Training Content

Training to become an AP was the most wished-for support (*n*=12). Participants emphasized the need to keep training simple and flexible, given the personalized nature of the service provided. Participants insisted that experiential knowledge cannot be learned in a training course. Instead, training should include a component on how the healthcare institution works and what names and acronyms are used in the unit to ensure smooth integration. In addition, know-how could be learned by highlighting the ability to ask open-ended questions and to be an active listener. They also mentioned the need to manage differing opinions on the best treatments or cultural differences. Personal abilities could also be addressed to share the fundamental skills and values of APs’ role and help potential APs determine if this role is proper.

### Reflecting on Remuneration and Professionalization

Participants expressed divergent views on the issue of remuneration. While few were adamantly opposed, others framed the discussion in relation to the impact of disease, loss of income, and the value placed on experiential knowledge. Some participants pointed out that illness had limited their reintegration into the workplace, for example, by affecting their ability to concentrate, and that remunerated AP work could represent an alternative source of income. One participant reported taking unpaid days off work to accompany patients and emphasised that financial compensation would mitigate such personal costs. Others argued that remuneration would signal greater recognition of experiential expertise and counter perceptions of APs as “amateurs” compared with salaried staff.

However, some participants rejected the professionalization associated with remuneration, explaining that it would compromise their subjectivity and freedom. In their view, this approach would lead to unionization, compulsory training, and practice standardization. Several emphasized the tension between altruism and financial gain, viewing accompaniment as a vocation that should remain disinterested and free from monetary obligation. From this perspective, remuneration was seen as incompatible with the underlying philosophy of the role.

### Reflecting on Institutionalization

Despite some participants’ reservations, the majority supported institutionalization of the AP role to ensure sustainability (n=10). With the exception of one, participants underscored the importance of endorsement by senior management. They noted that projects initiated at the unit level lacked equity, as patients’ access to AP services was dependent on whether individual providers chose to refer them. Institutionalization was therefore regarded as necessary for transparency, strategic planning, and equitable deployment of AP services across settings.

One participant further observed that institutionalization would enhance the legitimacy of AP contributions. They noted that while doctors could not ignore a note from a nurse, they could ignore notes from APs since APs were considered volunteers. Others expressed frustration with initiatives that did not come to fruition, suggesting that institutionalization would enable transparency and the development of strategies for deploying their functions on a larger scale.

Analysis of the data by gender, age, and type of disease or health condition showed no significant difference in perspectives on institutionalization.

## Discussion

This research was two-fold. First, it highlighted the ethical foundations of the AP role, from personal motivation to patient interaction to integration within clinical teams and healthcare institutions. It highlighted the values and skills needed to become an AP and their ongoing need for institutional integration.

We found that APs were selected for their personal qualities and skills in working directly with patients. The selection process is the first step in ensuring the quality of services in healthcare institutions by screening potential APs according to the qualities needed in a helping relationship.

Most of our participants have been involved as APs working alongside patients for many years, demonstrating their commitment and sincerity in developing this emerging role, even without monetary gain. They vouch for the sustainability and broader deployment of this service. The needs identified by our sample reveal the responsibilities of the healthcare system for promoting their services and protecting their well-being.

Our results showed an ethical tension between the independence of APs and the institutionalization of their role, which we define as establishing new markers in an existing organization for sustainability. APs reflected on institutionalization, professionalization, and remuneration impacting their independence. They interrogated the feasibility of aligning their role’s foundations within healthcare institutions, raising questions about unionization, compulsory training, and practice standardization. However, many of the facilitators identified by APs—such as recognition, coordination, and integration—are achievable and sustainable only through institutionalization.

## The Ethical Foundations of the Role of APs

### Experiential Knowledge: Core Skill of APs

All participants reported that experiential knowledge is irreplaceable. One must experience a disease or health condition to become an AP. This expertise differs from the therapeutic expertise of healthcare providers: the approach is more humanistic than medical (Ferville et al. 2023; Gabitova and Burke 2014; Walde et al. 2023), and is based on emotional, experiential, and social support (Shankland et al. 2022; Pomey, Paquette, et al. 2023; Pomey, Nelea, et al. 2023; Vigneault et al. 2015; Vahdat et al. 2014; Krist et al. 2017). This experiential knowledge gives them empathy and credibility, facilitating a relationship with the accompanied patient.

### Building a Supportive Relationship Based on Trust

Previous studies have highlighted that this unique relationship, sparked by experiential proximity, is based on foundations of authenticity, altruism, and humanity. This experiential proximity—sharing similar healthcare experiences and challenges in navigating through healthcare institutions—supports patients and increases their trust in the healthcare system. This trust is built not only from authenticity but also from APs’ neutrality regarding treatment decisions and their relative distance from institutional authority.

Participants emphasized the significance of personalizing their interventions with accompanied patients. They highlighted that their primary role was to promote patient partnership, integrating patients’ social circumstances into their care pathways to achieve better health outcomes (Dumez and Pomey 2019; Gates and Akabas 2007). In this way, APs allow patients to identify their concerns, preferences, and needs and empower them to discuss their therapeutic choices with healthcare providers.

An original contribution of our study lies in identifying honesty as an explicit value in this relationship. Participants report that honesty is important for establishing trusting relationships with their patients and is expressed along with a neutral and positive attitude of respect for and openness to the patients’ decisions.

APs also reported that reassurance is a central function of their role, echoing earlier studies (Richards et al. 2023; Pomey, Nelea, et al. 2023; Pomey, Paquette, et al. 2023; Vigneault et al. 2015; Ferville et al. 2023; Clavel and Pomey 2020; Abelson et al. 2022). Evidence suggests that patients who meet APs are more open to treatment options (Vigneault et al. 2015; Walde et al. 2023), feel less anxious (Pomey, Nelea, et al. 2023; Pomey, Paquette, et al. 2023), and have reduced requests for psychological counselling (Ferville et al. 2023). APs demonstrate to patients the possibility of returning to everyday life during or after episodes of care (Pomey, Paquette, et al. 2023). In this sense, APs occupy a unique role that builds patient confidence and empowerment.

### Exploring Hope and Distance: Nuance From Our Cohort

Our results reveal the nuances of two dimensions commonly described in the literature: hope and distance. Several studies have highlighted that APs function as models of hope (Shankland et al. 2022). Our APs revealed divergent responses. Half the participants saw hope as central to having a positive attitude and sharing strength and energy. Some individuals, however, express caution towards the concept of hope— it may not always exist or apply to a specific health situation. These APs stress that hope must not be understood as life without disease or health conditions. They stressed the importance of acceptance and resilience.

Similarly, research has often stressed the necessity of distancing oneself from one’s own illness experience to support others (Shankland et al. 2022). By contrast, APs in our study expressed mixed views, which may reflect the diversity of diseases and conditions of APs and patients. APs with chronic conditions cannot distance themselves from their care episodes, as their treatment is often ongoing, and in any case, the empathy valued by most participants implies some connection to the AP’s own clinical experience.

## Independence Versus Institutionalization Tension

APs emphasized the need to be independent to strengthen patients’ trust in the healthcare system and to preserve their disinterested posture regarding patients’ treatment preferences. However, independence may be illusory because APs collaborate with the clinical team to engage patients in their care, and they share common objectives with and expect support and recognition from healthcare institutions.

Pomey, Nelea, et al. (2023) have shown that the expansion of a national programme is supported by 86.8 per cent of accompanied patients after a first appointment and rises to 97.3 per cent after a second appointment, demonstrating the needs associated with and the success of this innovative role. Institutionalization will make it possible to measure the effectiveness of AP services on a larger scale, the benefits of which have been demonstrated and which will ultimately enhance the population’s health.

From that perspective, healthcare institutions should recognize their responsibilities toward APs. Because of the nature of their one-on-one intervention with patients, it must be recognized that they are different from other volunteers who perform more administrative and operational functions. APs bring unique and essential knowledge to improve the quality of care, and we must admit that this can challenge their mental health (Richards et al. 2023). In this respect, if patients are to benefit from the services of APs, healthcare institutions must assume their responsibilities regarding their integration, well-being, and safety.

We note a tension between the foundations of the APs’ role and the large-scale deployment of an AP programme to improve population health. We have used Tannahill’s framework background for ethical analysis in public health to propose an ethical reading of the tension according to the philosophical core of patient-partnership.

### Support Needed and Sense of Belonging: Experiences of Loyalty Conflicts

APs expressed a need for support and for a sense of belonging. They turned to healthcare organizations for guidance when ethical or relational challenges arose, demonstrating their affiliation, sense of responsibility, and perception of reliance on healthcare institutions and clinical teams. In one case, the boundaries between a friendly and professional relationship were crossed by a patient, and the participant turned to the clinical team for guidance on how to manage the situation so as not to undermine the patient’s adherence to their care. The absence of such guidance has the potential to result in APs crossing their personal space and disengaging from the healthcare system. In another case, one participant witnessed an injustice experienced by a patient and encouraged the patient’s family to fill out a complaint for negligent care, thereby affirming loyalty to institutional structures while upholding patient advocacy.

### Recognition of Expertise Versus Truthiness: The Monetary Gain Discussion

Recognition of experiential knowledge as expertise is well documented. Abelson et al. (2022) found that 61.7 per cent of their patient partners sample endorsed this view. Some participants in our sample questioned the valuation of this knowledge if they were the only ones working without remuneration. Our sample reported that they had received compensation to cover certain expenses (e.g., meals and parking). Some noted inequities and inefficiencies in compensation, such as lack of benefit from parking vouchers for those travelling by public transport, and proposed that healthcare institutions change this compensation for a flat rate to address disparities and acknowledge the unique value of AP knowledge, expertise and contribution to patient care.

One participant reported problems in retaining good APs. We suggest that offering a flat rate for AP contributions may retain competent APs in healthcare institutions.

We acknowledge the possibility of misuse—a malicious person who only wishes to gain this flat rate. However, the typical selection process (Clavel and Pomey 2020; Armstrong et al. 2013; Tritter and McCallum 2006) is sufficient to prevent such a situation and preserve the altruistic nature of the role. Currently, APs in Québec are recruited mainly by their treating providers and are volunteers. Despite lack of payment, we still see enthusiasm for this role. Because of the well-established selection process, flat rate attractiveness is secondary since APs already go through many administrative steps before encountering patients.

### APs’ Professional Autonomy

We argue that APs are partially professionalized because their activities are the culmination of activities performed by peer support groups, only in a structured practice, which can only be fulfilled by those with specific skills. Professionalization is separate from institutionalization because it concerns the interiority of a group practicing the same specialized activities.

Although the participants in our study clearly expressed the need to maintain flexibility and independence, several of the skills and values identified are recurrent among participants, allowing us to argue that there is a common foundation of the practice.

To this end, the central requirement in terms of skill—as unanimously identified in our study and widely recognized in the literature—is experiential knowledge (Karazivan et al. 2015; Dumez and Pomey 2019; Shankland et al. 2022; Vigneault et al. 2015; Montreuil, Martineau, and Racine 2019). Becoming an AP is impossible without lived experience with an illness or health condition. In this sense, the AP is not a conventional profession, in that the expertise is inherently subjective. AP flexibility and independence are essential to effectively accompany patients (Pomey, Nelea, et al. 2023) and necessary to adapt interventions to patients’ needs. APs occupy a partially professional space, separate from a full professionalization by the impossibility of limiting or generalizing the uniqueness of experiential knowledge into a consistent and uniform profession.

On this basis, developing a code of values emphasizing the basic principles of accompaniment, particularly altruism and empowerment, and reaffirming the foundations of the patient partnership without interfering in the independence of the relationship. This code could complement technical training (e.g., medical jargon, acronyms) for APs (Richards et al. 2023; Pomey et al. 2018; Fancott et al. 2018) and align with existing recommendations for successful partnership at all levels of governance (Pomey et al. 2016).

### No Harm: Accountability to Quality of Care and Responsibilities Toward APs

Unlike other patient partners, who are involved on educational, research, or governance levels, APs work directly with patients, heightening institutional accountability for their actions. Formalizing requirements ensures reliability and mitigates risks of harm, protecting both patients and institutions. APs, in turn, represent institutional values—excellence, benevolence, non-maleficence, autonomy and justice.

Our results show that APs require support, such as a resource person to coordinate their activities (Richards et al. 2023; Ferville et al. 2023; Clavel and Pomey 2020), psychological support after challenging encounters, and structured meetings between APs to debrief (Pomey, Paquette, et al. 2023; Ferville et al. 2023). In their cross-sectional survey working across healthcare systems in Canada, Abelson et al. (2022) report that 55.1 per cent of patient partners express a need for support from healthcare institutions. Institutionalization would codify healthcare systems’ obligations toward APs’ well-being and safety—conditions essential for retention and service sustainability.

### Equitable Services to the Population

Deployment of this role requires healthcare institutions and clinical teams to promote their services to patients, which is necessary for their activities to be known (Ferville et al. 2023) in an equitable access to services perspective. Being included as a full member and, most importantly, being recognized as a team member are critical facilitators identified by APs (Ferville et al. 2023). Services offered on the same unit, or even throughout one institution, necessarily require promotion from upper management, which goes along with a clear institutionalization (Ferville et al. 2023; Clavel and Pomey 2020). If healthcare providers do not inform patients about APs’ services the same way they present other services (e.g., access to a psychologist), the services are unequally offered among patients. Therefore, the accessibility of services should not be based on the knowledge or esteem of healthcare providers who meet patients (Ferville et al. 2023) but on equity and distributive justice for enhancing population health.

### The Cost of Losing Independence

Nonetheless, it must be acknowledged that APs give up some of their professional freedom in exchange for limited institutional control. However, this trade-off is acceptable insofar as institutional values—those that first inspire AP engagement remain central. Desire to give back to the system motivates APs to get involved (Pomey, Paquette, et al. 2023; Vigneault et al. 2015; Ferville et al. 2023; Abelson et al. 2022). One-on-one contact with patients means that APs take responsibility for their actions and are bound by their institution’s quality of care standards. Partial professionalization could give them institutional recognition of their expertise, which reduces the unequal relationship between healthcare providers and APs. Experiential knowledge should remain central in regulating AP practice, provided it operates within legal and professional limits and in collaboration with healthcare teams. This balance preserves the distinctiveness of APs while integrating them into institutional frameworks.

This proposed framework for APs is necessary to ensure equitable access to patient support services. Up to now APs have been maintained by research funds, which is not sustainable. Studies have shown that poor operationalization of partnership activities results in structural and philosophical problems (Liang et al. 2018; Martin and Finn 2011; Moore and Prentice 2015). To ensure continuity of service, institutionalizing AP services and the involvement of managers are imperative (Clavel and Pomey 2020; Alexander and Hearld 2011). To achieve this, institutions must assume responsibilities for APs, provide favourable practice conditions, and avoid interfering with their interventions so that practice remains rich and beneficial.

The implementation of this role means committing to population health and the democratization of health. Collaboration must be prioritized in negotiations between decision-makers and APs to cultivate a robust partnership. In order to pursue innovation in practice, care solutions adapted to different units should be negotiated with APs, centralizing their experiential knowledge.

APs stated that their distance from the institution fosters patient. The most significant risk associated with institutionalization is the potential for organizational and political interests of the institution to override APs priorities, ultimately losing track of the philosophical core of the patient partnership. On the one hand, institutionalization addresses concerns regarding acknowledgement of AP competencies and contribution to the healthcare system. Moreover, institutionalization helps guarantee the consistent quality of services and equitable distribution of healthcare to the population. On the other hand, the partial loss of independence inherent in institutionalization, can influence AP self-perception—and the perception of others—of distance from the institution. This can mitigated by an APs’ unbiased approach, active listening, and experiential proximity. On balance, our analysis supports the greater benefits to be gained through institutionalization and the capabilities of APs to fulfil their role with both institutional affiliation and independent integrity as integral clinical team members.

## Limitations

Our study has limitations. First, participants were recruited in the circle of our research team. The recruitment of participants may have influenced our results. Second, we have disaggregated data by gender, age, and type of disease or health condition, meaning these data must be used cautiously. Our study does not allow us to identify, confirm, or refute trends according to sociodemographic characteristics. A quantitative gender-based study on the background of APs would be necessary to deepen these questions.

## Conclusion

APs are former patients who support other patients in their care journeys. This partnership approach embodies peer support and the philosophy of empowerment. Our participant’s essential skills and values align with this partnership philosophy, comprising first-hand experience, attentive listening, empowerment, respect and altruism (Shankland et al. 2022).

Participants in our study identified several foundations essential to their role: their subjective experience living with disease or health conditions, altruism towards other patients, genuine empathy, and openness to care choices. These provide a common ground for the ethical foundations of AP work.

This study revealed an ethical tension between the independence of APs and the need for institutionalization. Addressing the goals of the patient partnership philosophy and based on a public health ethics analysis, we find that the institutionalization of APs allows them to act with sufficient independence with the potential to enhance the population’s health. By using a lens of public health ethics, we find that achieving independence and authenticity as an AP is not necessarily incompatible with institutionalization, partial professionalization, and remuneration. The institutionalization of APs, however, must be aligned with the philosophy of patient partnership, remain self-regulated, and framed according to flexible criteria.

## Supplementary Information

Below is the link to the electronic supplementary material.

Supplementary file1 (DOCX 23.3 KB)

## Data Availability

Data are not available because participants did not consent to data sharing. The interview guide is available as an additional material to this publication.
