# Supplementary material for: The Ethical Foundations of Accompanying Patients: The Need for Institutionalization
Source: J Bioeth Inq. 2025 Dec 1;23(2):353–75. doi: 10.1007/s11673-025-10482-z (PMC13388418; doi:10.1007/s11673-025-10482-z)
Supplement: Supplementary file 1 — Supplementary file1 (DOCX 23.3 KB) [file 11673_2025_10482_MOESM1_ESM.docx]

**Interview guide**

**Project: Ethical Foundation of the Role of Accompanying Patient**

Introduction and background :

1. How long have you been an accompanying patient?
2. How did you become an accompanying patient?
3. Are you part of a group as an accompanying patient, such as an establishment, institute or clinical department?

Personal values

1. Why did you choose to become an accompanying patient?
2. What motivated you to pursue your role?
3. What do you find most important in this role?
4. What do you think it takes to become an accompanying patient?
   1. What are the attitudes and behaviors you should adopt as an accompanying patient?

Accompanying patient status

1. Should the accompanying patient have an officially recognized status?
2. If so, how should this status be recognized?
   1. Should the accompanying patient be paid or be a volunteer?
   2. What do you think would be a reasonable level of recognition or remuneration?
   3. Are there other ways in which the accompanying patient could gain recognition for his or her role?
3. In your opinion, what is the accompanying patient's current place within the system and/or organization?

Role within the care team

1. Do you feel part of the healthcare team?
2. Do you feel recognized by the healthcare team?
3. If you are part of the care team, what is your role in the team?
   1. Is your role defined in relation to other team members?
   2. Is your role well understood by other team members?
   3. How do you think other team members perceive your role?
   4. Have you ever had the impression of being instrumentalized in your duties by managers or health professionals? If so, in what way?
4. How would you characterize your experience with the healthcare team as an accompanying patient?
   1. Do you feel supported and able to get help with your interventions?
   2. Do you have or would you like to have meetings with the care team?

Accompanying patient into action

1. Think of situations in which you have been an accompanying patient:
   1. What were your strengths?
   2. Have you experienced any difficulties in your role? What difficulties?
      1. Have you ever had to end your support relationship? What happened?
      2. Have you encountered cultural and/or ideological barriers with the patient you are supporting?
2. How do you deal with your personal experience as a patient and your role as an accompanying patient?
   1. Did you need to step back from your own care episode before becoming an accompanying patient?
   2. Were you yourself accompanied when you were a patient?
3. What should (or should not) be the relationship between an accompanying patient and a patient?
4. How do you deal with the perception of being a model of living with illness, possibly a model of hope?

Meeting organization

1. In an ideal world, how would you like your relationship with your care team to be organized?
2. What should be the place of tele-accompaniment and/or telephone versus face-to-face meetings?
3. Have you ever been unable to accompany someone for organizational reasons? If so, why?

**Original language**

**Grille d’entretien**

**Projet : Les fondements éthiques du rôle de patient-accompagnateur**

Introduction et mise en contexte :

1. Depuis quand êtes-vous patient accompagnateur?
2. Comment êtes-vous devenu patient accompagnateur?
3. Faites-vous parti d’un regroupement en tant que patient-accompagnateur tel un établissement, un institut, un service clinique?

Valeurs personnelles

1. Pourquoi avez-vous choisi d’être un patient accompagnateur?
2. Qu’est-ce qui vous a motivé à poursuivre votre rôle?
3. Qu'est-ce que vous trouvez le plus important dans ce rôle?
4. Selon vous, qu’est-ce qu’il faut pour devenir un patient accompagnateur?
   1. Quelles sont les attitudes et comportements à privilégier en tant que patient accompagnateur?

Statut du patient accompagnateur

1. Est-ce que le patient accompagnateur devrait avoir un statut reconnu officiellement?
2. Si oui, de quelle manière ce statut-ci devrait-il être reconnu ?
   1. Le patient accompagnateur devrait-il être rémunéré ou agir en tant que bénévole?
   2. Qu’est-ce qui serait raisonnable comme reconnaissance ou rémunération selon vous?
   3. Il y a-t-il d’autres façons dont le patient accompagnateur pourrait obtenir de la reconnaissance pour son rôle?
3. Selon vous, quel est la place actuelle du patient accompagnateur au sein du système et/ou de l’organisation?

Rôle au sein de l’équipe soignante

1. Avez-vous le sentiment de faire partie de l'équipe soignante?
2. Avez-vous l'impression d'être reconnu par l'équipe soignante?
3. Si vous faite partie de l'équipe soignante, quel est votre rôle dans l'équipe?
   1. Est-ce que votre rôle est défini par rapport aux autres membres de l’équipe?
   2. Est-ce que votre rôle est bien compris par les autres membres de l’équipe ?
   3. Comment pensez-vous que les autres membres de l’équipe perçoivent votre rôle?
   4. Est-ce que vous avez déjà eu l’impression d’être instrumentalisé(e) dans vos fonctions par les gestionnaires ou professionnels de la santé? Si oui, de quelle manière?
4. Comment caractérisez-vous votre expérience avec l’équipe soignante en tant que patient accompagnateur?
   1. Vous sentez-vous épaulé(e) et sentez-vous que vous êtes en mesure d'avoir de l'aide en lien avec vos interventions?
   2. Avez-vous ou souhaitez-vous avoir des rencontres avec l’équipe soignante?

Mise en action du rôle de patient accompagnateur

1. Pensez à des situations où vous avez été patient-accompagnateur
   1. Quelles ont été vos forces?
   2. Avez-vous vécu des difficultés dans votre rôle? Lesquelles?
      1. Avez-vous déjà eu à mettre fin à votre relation d’accompagnement? Comment ça s’est passé?
      2. Avez-vous rencontré des barrières culturelles et/ou idéologiques avec le patient accompagné?
2. Comment gérez-vous votre expérience personnelle de patient et votre rôle de patient accompagnateur?
   1. Avez-vous eu besoin d’un recul face à votre propre épisode de soins avant de devenir patient-accompagnateur?
   2. Avez-vous vous-même été accompagné(e) lorsque vous étiez patient?
3. Quelle devrait (ou pas) être la relation entre un patient accompagnateur et un patient?
4. Comment gérez-vous la perception d’être un modèle de vécu avec la maladie, possiblement un modèle d’espoir?

L’organisation des rencontres

1. Dans un monde idéal, comment souhaiteriez-vous que s’organise la relation avec l’équipe soignante?
2. Quelle devrait être la place du télé-accompagnement et/ou le téléphone versus les rencontres en personne?
3. Est-ce que cela vous est déjà arrivé de ne pas pouvoir accompagner une personne pour des raisons d’organisation ? Si oui, pourquoi?
